# 9α,11β-PGF_2_, a Prostaglandin D_2_ Metabolite, as a Marker of Mast Cell Activation in Bee Venom-Allergic Patients

**DOI:** 10.1007/s00005-015-0334-1

**Published:** 2015-03-13

**Authors:** Marita Nittner-Marszalska, Ewa Cichocka-Jarosz, Marek Sanak, Magdalena Wujczyk, Anna Dor-Wojnarowska, Grzegorz Lis, Jerzy Liebhart

**Affiliations:** 1 Department of Internal Diseases, Geriatrics and Allergology Medical University, Wroclaw, Poland; 2 Department of Pediatrics, Jagiellonian University Medical College, Cracow, Poland; 3 Department of Medicine, Jagiellonian University Medical College, Cracow, Poland

**Keywords:** PGD_2_, 9α,11β-PGF_2_, Skin chamber method, Bee venom provocation

## Abstract

Mast cell (MC) mediators, among them prostaglandin D_2_ (PGD_2_) and 9α,11β-PGF_2_, PGD_2_’s metabolite, play a key role in allergic reactions, including bee venom anaphylaxis (BVA). Assessment of these mediators has never been performed in BVA. The aim of the study was to assess the activation of MC during in vivo provocation with bee venom (BV) and to measure PGD_2_ and 9α,11β-PGF_2_ in the course of an allergen challenge. The second aim was to determine if assessment of these mediators could be useful for predicting adverse events during venom immunotherapy (VIT). In 16 BV-VIT patients and 12 healthy subjects, levels of PGD_2_ and 9α,11β-PGF_2_ were assessed during BV provocation by means of the skin chamber method. Chamber fluids, collected at 5 and 15 min, were analyzed for both mediators by gas chromatography mass spectrometry negative ion chemical ionization. BVA in comparison to non-allergic patients had a significantly higher ratio of 9α,11β-PGF_2_ in allergen-challenged chambers to 9α,11β-PGF_2_ in allergen-free chambers after 15 min of provocation (*p* = 0.039). Allergen challenge resulted in a significant increase of 9α,11β-PGF_2_ levels between 5 and 15 min after provocation only in BVA patients (*p* < 0.05). Analysis of log-transformed PGD2 levels showed significant difference between changes in PGD_2_ concentration between BVA and healthy subjects. No study patient developed adverse reactions during. 9α,11β-PGF_2_ is actively generated during the early allergic response to BV. Skin chamber seems to be a promising, non-invasive and safe model of in vivo allergen provocation in BV-allergic patients. High or low levels of both mediators do not predict occurrence of adverse events during VIT.

## Introduction

Severe systemic allergic reactions to bee venom (BV) are potentially life-threatening events, which makes this type of allergy a serious clinical problem (Antonicelli et al. 2002; Bilo 2011). Clinical manifestation is caused by a sudden release of mediators derived from effector cells, with mast cell (MC) mediators playing the most relevant role. Nowadays, specific IgE-based measurements: intradermal tests (IDT) with venom extract and specific serum IgE (sIgE) detected with ultrasensitive immunocapture methods are the main tool in the diagnosis of patients with the history of systemic reactions to bee sting (Tracy et al. 2011). However, these diagnostic tools can neither adequately predict severity of future sting reactions nor serve to monitor clinical reactivity to insect sting. Therefore, there is still research going on to work out clinically relevant markers of venom anaphylaxis and immunoresponse to venom immunotherapy (VIT).

One of the research paths has been seeking markers of the reactivity of MC cells that could be useful in both the diagnosis and the monitoring of the management of BV allergy. Among in vitro detectable MC-derived mediators, only serum tryptase has an established position in the diagnosis of venom anaphylaxis both in adults and children (Brown et al. 2013; Ruëff et al. 2009).

Another MC-derived mediator—prostaglandin D_2_ (PGD_2_)—a major cyclooxygenase product is released predominantly by activated MC, but also basophils (Ugajin et al. 2011). As a relatively unstable molecule, it is readily metabolized by NADPH-dependent 11-ketoreductase to 9α,11β-PGF_2_, a prostanoid of similar biochemical properties and potency as PGD_2_ (Beasley et al. 1987). Till now detection of PGD_2_ metabolites in blood, urine samples or exhaled air condensates has been found to be useful in monitoring both asthmatic adults and children (Bochenek et al. 2004; O’Sullivan et al. 1998; Sanak et al. 2011; Ono et al. 2009) were the first ones who proved that urinary concentration of 9α,11β-PGF_2_ is a reliable marker of endogenous production of inflammatory mediators within the natural course of anaphylaxis or during positive challenge tests (Higashi et al. 2010). There are also preliminary data on monitoring blood plasma and urine concentrations of PGD_2_ derivates in adults and children treated with specific immunotherapy (Cichocka-Jarosz et al. 2011, 2014; Rank et al. 2013).

Tangentially to Ono’s line of research, we made an attempt to estimate the usefulness of PGD_2_ and 9α,11β-PGF_2_ measurements as markers of bee venom allergy (BVA) during skin provocation with BV extract, comparing BV-allergic patients with healthy, venom non-allergic subjects. We used the skin chamber technique which is a safe and easily available model of in vivo estimation of MC activation.

The aim of the study was to assess the activation of MC during in vivo provocation with BV and to measure PGD_2_ and 9α,11β-PGF_2_ in the course of an allergen challenge. The second aim was to determine if assessment of these mediators could be useful for the assessment of MCs activation and predicting the clinical response of BV-allergic patients to VIT.

## Materials and Methods

### Subjects

Patients were recruited from the Department of Internal Diseases, Allergology and Geriatrics, Medical University of Wroclaw, Poland, EU.

The study subjects formed two groups: the BV-allergic group and healthy, non-BV-allergic controls. The BV-allergic group consisted of 16 untreated with VIT patients selected according to the following criteria: (1) a history of severe allergic reactions (III°/IV° according to Mueller’s classification) after a bee sting, and (2) positive results of IDT with BV extract at the concentration of 10^−6^ g/l. The control group consisted of 12 healthy, non-allergic patients, with no history of insect venom allergy and no sensitization to BV, which was confirmed by negative results of IDT with BV extracts in the concentration of 10^−3^g/l. The characteristics of the study groups are presented in Table 1. No study subject was on a medication interfering with the test performed (as topical and systemic corticosteroids, and antihistamine drugs). In the case of all the patients, ultra-rush (VIT-UR) method was applied. The method’s protocol assumes that the cumulative dose of 101.1 mcg of venom is reached within 3.5 h. Pharmalgen vaccine (Bee venom Pharmalgen, Alk-Abello, Denmark) was used throughout. VIT safety assessment was performed after 24 h since VIT-UR was initiated. The test was done 7–10 days before VIT. The safety assessment concerned the course of VIT-UR that lasted about 3.5 h and the period of 20 h after its completion.

**Table 1:** Characteristics of all studied subjects

	BV-allergic patients (*N* = 16)	Healthy subjects (*N* = 12)
Age (years)		
Mean (± SD)	38.81 ± 13.88	39.17 ± 18.53
Range	16–61	23–67
Me	34	29
Male/female	8/8	5/7
History of venom sting allergy	16/16 positive (III^o^ and IV^o^ according to Mueller)	12/12 negative
Positive/negative IDT with BV at the concentration of		
10^−6^g/l	16/16 positive	12/12 negative
10^−3^g/l	Not performed	12/12 negative
Bee venom sIgE (mean ± SD)	23.83 (±28.61) kU/l	Not performed

*Me* median, *IDT* intradermal test

The local Ethics Committee approved the study (KB 240/210). A written informed consent was obtained from each patient.

### Intradermal Tests

Lyophilized BV extract (Pharmalgen, Alk-Abello, Denmark) was used for skin testing. Skin tests were performed by intradermal injection of 0.02 ml solution at the concentration of 10^−6^ g/l (BVA group), up-dosing to maximum concentration of 10^−3^ g/l (healthy control group) with a positive (histamine dihydrochloride solution) and negative (saline) control according to the last recommendation of the European guidelines (Krishna et al. 2011). A wheal reaction of ≥5 mm in diameter was defined as positive. The test result was considered negative when the wheal reaction was <5 mm diameter after the injection of venom at the concentration of 10^−3^ g/l.

### Procedure of Venom Extract Provocation in the Skin Chambers

Skin chamber provocation test was performed according to the method described and used in the studies published by Zweimann et al. (1995) and Zweiman and von Allmen (2000). The test procedure was done in the four following steps:

1. The induction of blisters by heat and suction (a negative pressure suction system).
  The plastic chambers were placed on a cleaned skin area on the volar aspect of each forearm. The chambers were connected with a negative pressure cutaneous suction chamber system (Dermavac suction chamber unit, P. Bjerring, Marselisborg Hospital, Arrhus, Denmark). The skin blisters were induced with gentle heat (40 °C) and suction of 290 mmHg generated by this system. The process of suction was continued until complete blisters of approximately 8 mm in diameter were formed. The epidermal blister roofs were cut off at the skin level.
2. Fixing the skin chamber onto the volar side of a forearm.
  Transparent plastic chambers modeled on the chambers used in the Department of Physiology and Pharmacology, Karolinska Institute, Sweden, were placed over denuded blisters and secured with tape. The fixed chambers were rinsed four times with sterile phosphate buffered saline following which the wash fluid removed.
3. Administering allergen solution into the skin chamber.
  The allergen-challenged chamber was filled with BV extract (Bee venom, Pharmalgen, Alk-Abello, Denmark) at the concentration of 10^−4^ g/l and the total volume of 0.5 ml. The venom solution was always freshly prepared. The allergen-free chamber on the other arm was filled with venom diluent (human serum albumin 0.3 mg/ml in sodium chloride solution with 0.1 % phenol, Pharmalgen, Alk-Abello, Denmark).
4. Aspiration of the test fluid (allergen solution + exudation products) out of the chamber.
  After 5 min of incubation, the fluids from both chambers were harvested and collected into plastic tubes. Following this, the allergen-challenged chamber was refilled with BV extract at the same concentration (of 10^−4^ g/l in a total volume of 0.5 ml) and the allergen-free chamber was refilled with the diluent; thereafter, the test was continued for the next 10 min. Then, the fluids from the chambers were removed and collected into plastic tubes.

### Assessment of PGD_2_ and 9α,11β-PGF_2_ Concentrations

Fluid samples were immediately centrifuged at 3500 revolutions per minute for 10 min and then stored at −80 °C. They were assayed within 1 month. Before an assay, 0.5 ng of internal deuterated standards of PGD_2_ and PGF_2α_ [^2^H] PGF_2α_ (Cayman Chemicals, Ann Arbor, Michigan, USA) was added to 1 ml of sample fluid, aiming to compensate for the loss of the analyte during sample preparation.

Measurements of PGD_2_ and 9α,11β-PGF_2_ were performed using gas chromatography negative ion chemical ionization mass spectrometry (model 5896 series II; Hewlett Packard, Palo Alto, CA, USA) as described elsewhere (O’Sullivan et al. 1999). The diagnostic ions were 489 *m*/*z* for PGD_2_ and 495 *m*/*z* for internal standard; while for 9α,11β-PGF_2,_ they were 569 *m*/*z* and 573 *m*/*z*, respectively. The detection limit was 1 pg/ml, the concentration was expressed in picograms per milliliter (Bochenek et al. 2003).

### Statistical Analyses

Analyses were performed using Statistica 10.0 program. The data distribution was tested by Shapiro–Wilk *W* test. The correlation between both PGD_2_ and 9α,11β-PGF_2_ values in patients with BVA was assessed using Spearman rank analysis. Comparisons between groups were performed with the Mann–Whitney *U* test. Comparisons within the groups (pre- and post-challenge values) were analyzed by Wilcoxon test with “post hoc” analysis. Receiver operator characteristics (ROC) were applied for evaluation of usefulness of analyzed parameters for discrimination between BV-allergic and control group.

To assess independent predictors of PGD metabolites, both PGD_2_ and 9α,11β-PGF_2_ levels were log-transformed. As distributions of both log-transformed variables were still significantly different from normal, five subjects had to be excluded from BV-allergic group, as outliers, to fulfill conditions of use of Generalized Estimating Equations (GEE) model concerning distribution of dependent variable. Then, multivariate GEE model for dependent variable of normal distribution and identity linking function was used, taking into account: groups (BVA vs controls), time of measurement and type of chamber as categorical predictors. Values of *p* < 0.05 were considered to be significant.

## Results

### Clinical Observations

The characteristic of the study groups is presented in Table 1. All patients completed the study. During skin provocation with BV by means of skin chamber method, neither local nor systemic adverse effects were observed. No clinical symptoms of allergy or negative side effects after the test’s completion were noted. No late reactions were reported.

### Recovery of Skin Exudates

We recovered more than 90 % of fluid in all chambers with the exception of allergen-free chambers (due to leakage) in two BVA patients in whom we only analyzed the mediators’ concentrations in allergen-challenged chambers (allergen chambers).

### PGD_2_ and 9α,11β-PGF_2_ Values in Control Group

In healthy subjects, the levels of PGD_2_ and 9α,11β-PGF_2_ did not differ statistically between allergen-challenged and allergen-free chambers after 5 and 15 min of provocation (Table 2). No differences in 9α,11β PGF_2_ and PGD_2_ concentrations we found in at 5 and 15 min of incubation with the allergen.

**Table 2:** 9α,11β-PGF_2_ and PGD_2_ concentration in pg/ml (Me [min; max]) in the fluid from allergen-challenged chambers and allergen-free chambers in BV-allergic patients and control

Time/solution/release ratio	Control group	BV group	Control vs BV
9α,11β-PGF_2_ (pg/ml)			
5-min incubation			
BV solution	10.1 [2.8; 45.5]	4.9 [1; 1962.6]	*p* = 0.46
Control solution	6.3 [1.8; 30.8]	2.85 [0.9; 22.1]	*p* = 0.04
Release ratio (BV/control solution)	1.24 [0.44; 7.88]	2.15 [0.52; 222.17]	*p* = 0.26
15-min incubation			
BV solution	12.45 [3; 59]	27.3 [2; 711.8]	*p* = 0.3
Control solution	14.2 [3.2; 64.7]	6.8 [1.5; 48.9]	*p* = 0.08
Release ratio (BV/control solution)	0.8 [0.2; 164]	1.8 [0.5; 385]	*p* = 0.039
PGD_2_ (pg/ml)			
5-min incubation			
BV solution	181.4 [106; 536.8]	184.3 [18.2; 9710.6]	*p* = 0.71
Control solution	115.2 [35.7; 699.7]	122.3 [24.5; 490.3]	*p* = 0.95
Release ratio (BV/control solution)	2.56 [0.18; 5.36]	1.48 [0.19; 21.19]	*p* = 0.79
15-min incubation			
BV solution	140.2 [22.8; 299.4]	314.3 [17.5; 3855.3]	*p* = 0.23
Control solution	142.85 [51.7; 315]	169.7 [24.8;746.2]	*p* = 0.4
Release ratio (BV/control solution)	1.17 [0.11; 2.04]	1.31 [0.09;27.47]	*p* = 0.54

*Release ratio* ratio of the mediator levels in the fluid chamber after BV incubation solution to the control solution for given patient and time incubation

### PGD_2_ and 9α,11β-PGF_2_ Values in BV-Allergic Group

Using whole group comparisons, no significant differences of the concentrations of PGD_2_ between allergen-challenged chambers and allergen-free chambers were noted at either time point, including 5- and 15-min provocations.

Although in BV-allergic patients the level of 9α,11β-PGF_2_ was higher after 5 (2 times) and 15 min (4.5 times) of incubation in the allergen-challenged chambers than in allergen-free chambers, these differences did not achieve statistical significance.

**Fig. 1:**
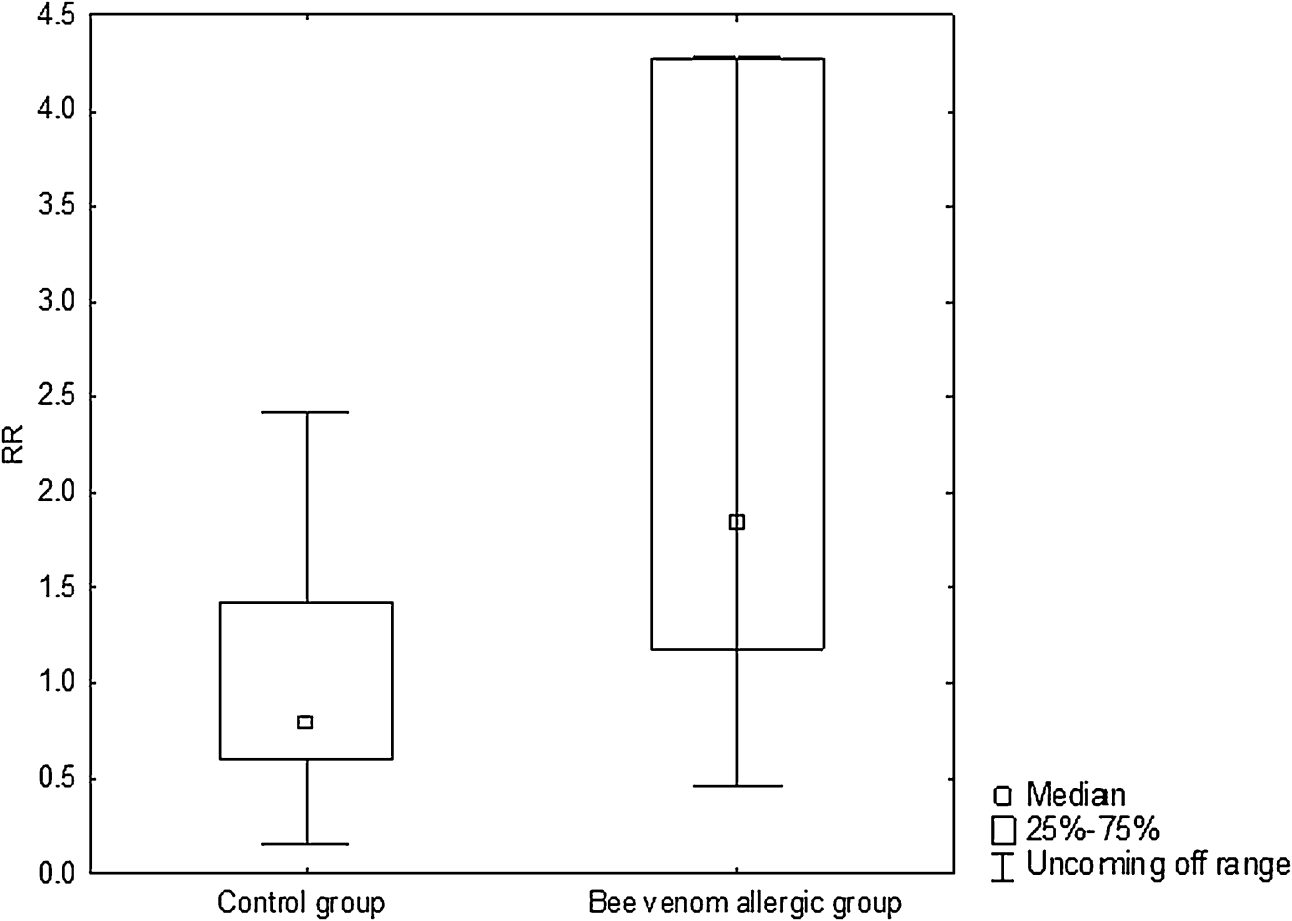
The difference between control group and BV-allergic group in 9α,11β-PGF_2_ value after 15 min of allergen provocation (the value of 9α,11β-PGF_2_ after 15 min in allergen-challenged chambers/9α,11β-PGF_2_ after 15 min in allergen-free chambers). Release Ratio (RR): ratio of the mediator levels in the fluid chamber after BV incubation solution to the control solution for given patient and time incubation

In comparison with healthy subjects, BV-allergic patients demonstrated significantly higher release of 9α,11β-PGF_2_ in allergen-challenged in comparison with allergen-free chambers after 15 min of provocation *p* = 0.039 (Fig. 1; Table 2). Only BV-allergic patients presented also a significant increase in 9α,11β-PGF_2_ concentrations between 5 and 15 min of provocation with allergen.

**Fig. 2:**
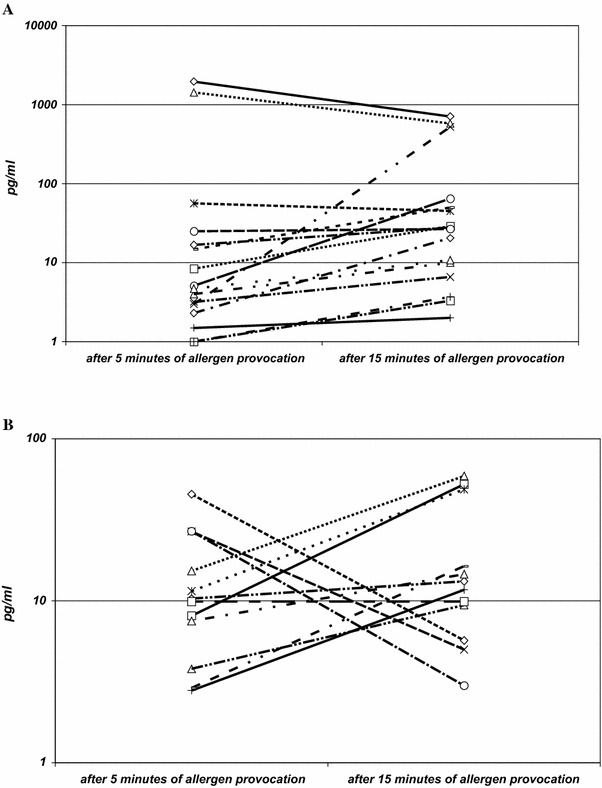
**a** Individual response of 9α,11β-PGF_2_ in BV-allergic group (16 venom-allergic subjects) after 5 and 15 min provocation with allergen. **b** Individual response of 9α,11β-PGF_2_ in bee venom insensitive group (12 venom non-allergic subjects) after 5 and 15 min provocation with allergen

The reactions to BV extract in BV group were heterogeneous: an increase of 9α,11β-PGF_2_ (1.07–174-fold) was observed in 12 patients, while a decrease (1.2- to 2.7-fold) was noticed in four patients (Fig. 2a). Striking was an extremely high dispersion of results obtained after challenge with allergenic extract in BV group (Table 2). In two patients, the values of 9α,11β-PGF_2_ after 5 min of provocation exceeded 1400 pg/ml and after 15 min exceeded 524 pg/ml; while in other BV-sensitive patients, they were lower than 62 pg/ml.

### Multivariate Analysis

Application of multivariate analysis showed that log-transformed PGD_2_ level increased significantly in BVA group between 5 and 15 min after provocation (*p* = 0.025); while in control group, we observed non-significant decrease—the difference between changes observed in both groups was statistically significant (*p* = 0.010), We observed decrease in log-transformed level of PGD_2_ in allergen-challenged chamber, opposite to increase observed in diluent chambers, but this difference did not reached statistical significance (*p* = 0.071).

In case of log-transformed 9α,11β-PGF_2,_ its levels significantly increased between 5 and 15 min after provocation (*p* < 0.001) and were higher in challenged chambers (*p* = 0.007). Level of log-transformed 9α,11β-PGF_2_ concentration was lower in BVA group; however, this difference was of borderline significance (*p* = 0.056).

### ROC Curve

**Fig. 3:**
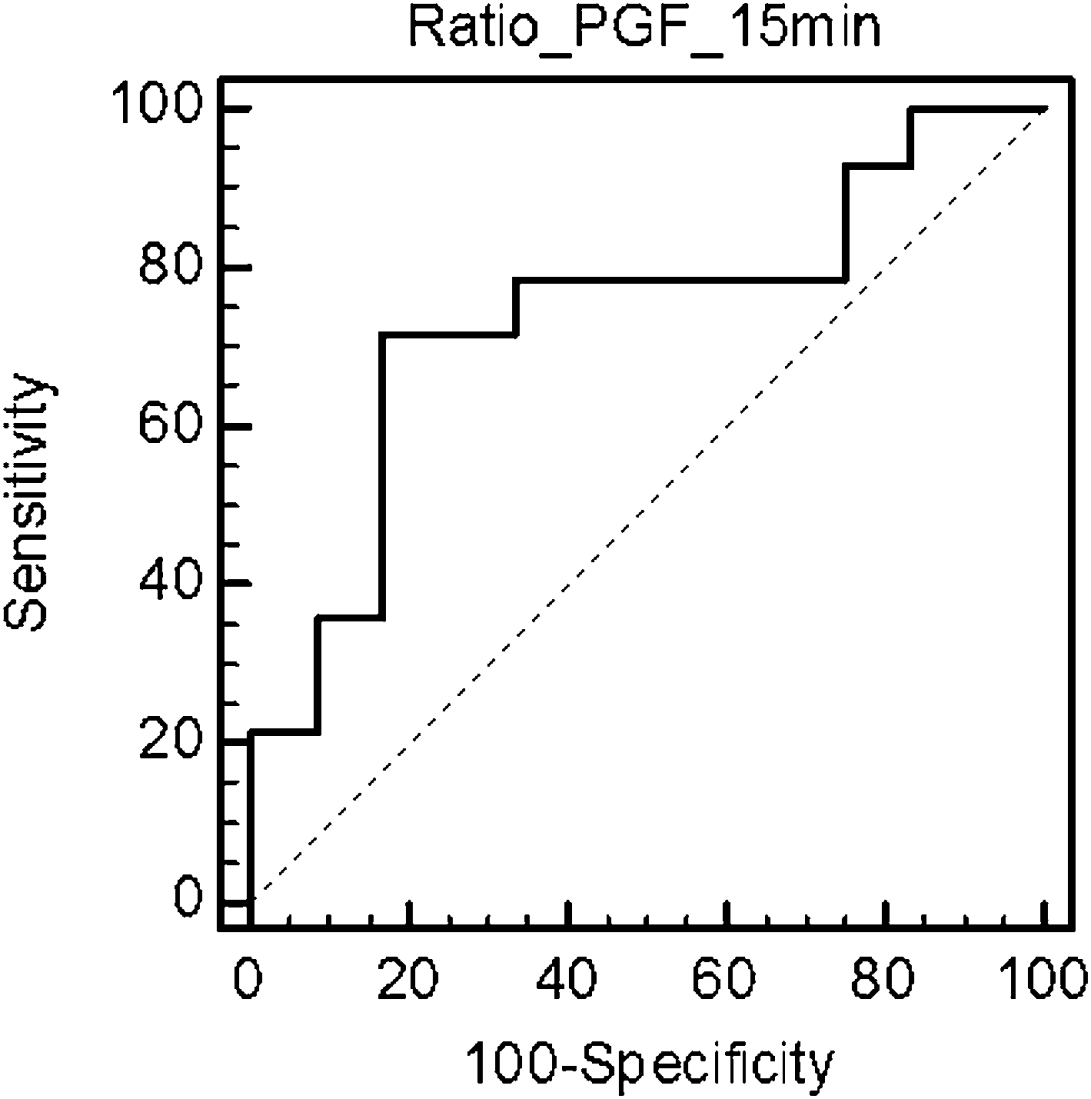
ROC curve for ratio of 9α,11β-PGF_2_ concentration between 15th and 5th minute in allergen-challenged chamber

To evaluate usefulness of 9α,11β-PGF_2_ and PGD_2_ ratios after allergen extract provocation in discrimination between allergic and control group ROC curve was applied (Fig. 3). Only a parameter calculated as fold change (ratio) of 9α,11β-PGF_2_ concentration between 15th and 5th minute in allergen-challenged chamber revealed a significant area under ROC curve (AUC = 0.74; *p* = 0.019). For the ratio equal to 1.62, the optimal sensitivity (equal 71.4 %) and specificity (equal 83.3 %) were observed.

### Correlation Analyses

Only in the BV-allergic group, there were significant correlations between the levels of 9α,11β-PGF_2_ at 5 and 15 min of provocation (Spearman’s *ρ* = 0.75; *p* = 0.0007) as well as between the levels of PGD_2_ at 5 and 15 min of provocation (*ρ* = 0.62; *p* = 0.01). A high correlation coefficient was found in the BV-allergic group between 9α,11β-PGF_2_ and PGD_2_ levels at 5 and 15 min, respectively (*ρ* = 0.8; *p* = 0.0001 and *ρ* = 0.9; *p* = 0.000002). No correlation was found between sIgE levels and measured prostanoids.

### Allergen Immunotherapy

No patient developed symptoms of adverse reactions during allergen immunotherapy.

## Discussion

Among various MC mediators involved in allergy and asthma pathophysiology, derivates of arachidonic acid take a special place. So far, cyclooxygenase metabolite has been intensely investigated in aspirin-induced asthma (Antczak et al. 2002; Celejewska-Wójcik et al. 2012; Higashi et al. 2002; Mastalerz et al. 2012).

From the point of view of our investigation, especially important are studies assessing the production of PGD_2_ and its metabolites after provocation with allergens (Sood et al. 2013). Urinary 9a,11b-PGF_2_, as a metabolite related to MC activation, was previously suggested by Ono et al. (2009) as a sensitive marker of anaphylactic reaction. Bochenek et al. (2004) suggested that in asthmatic adults, plasma 9a,11β-PGF_2_ may be a sensitive measurement in detecting MC activation during bronchial challenge with allergens. Both O’Sullivan et al. (1998) and Mita et al. (2001) reported on increased urinary 9α,11β-PGF_2_ levels after aspirin challenge in aspirin-sensitive patients, whereas Sladek et al. (1991) reported an early 3.8-fold rise in urinary levels of another PGD_2_ metabolite, tetranor PGD-M. Also Bochenek et al. (2003) using gas chromatography/mass spectrometry measurements, showed that acetylsalicylic acid (ASA) provocation caused early 1.4-fold rise of 9α,11β-PGF_2_ in blood of ASA-sensitive patients in comparison with the pre-challenge values. The similar results were obtained in asthmatic patients sensitized to *Dermatophagoides pteronyssinus* or grass allergens in whom bronchial challenge with specific allergen resulted in an increase in the mean plasma and urinary 9α,11β-PGF_2_ levels within 2 h after provocation (Bochenek et al. 2004).

The main aim of this study was to assess the release of PGD_2_ and 9α,11β-PGF_2_ after an in vivo challenge with the specific allergen in subjects with BVA, using the skin chamber method. We showed that during provocation of the epiderm-denuded skin surface with specific allergen in BV-allergic patients, there occurs an increase in the release of 9α,11β-PGF_2_ between 5 and 15 min time points of challenge. In BV-allergic patients, mean 9α,11β-PGF_2_ concentration after 15 min provocation with allergen increased fivefold as compared with the 9α,11β-PGF_2_ after 5-min antigen provocation (1.07- to 174-fold in particular patients), and tenfold as compared with the baseline values measured in the control skin chambers filled with diluent alone. In healthy non-allergic patients, no marked increase in 9α,11β-PGF_2_ release in the course of allergen provocations was noticed. The factor distinguishing BV-allergic patients from control patients is a dynamic increase in 9α,11β-PGF_2_ release after allergen provocation.

The novelty of our study consists in demonstration for the first time, that 9α,11β-PGF_2_ and PGD_2_ are released from the skin MC in venom-allergic patients upon topical challenge. Since these mediators are measured directly, a variable vascular reactivity, which is required for IDT readout, is avoided. Additionally, the method of in vivo skin chamber that we used has a capability for other studies investigating of MC activation processes. In few studies, this method was used to investigate mediator release and cell response during developing early and late phase of cutaneous allergic reactions (Fernvik et al. 1999a, b; Nopp et al. 2000). Only in two published studies known to us, PGD_2_ was assessed in atopic patients after skin provocation with allergen using the same method of the skin chamber technique. In the study conducted by Atkins et al. (1990), the authors demonstrated PGD_2_ release in atopic subjects in the first hour of allergen provocation. Pienkowski et al. (1988), using the skin blister technique, reported post-provocation peak level release of PGD_2_ in ragweed allergic patients after 2.5 h. Because our timings were different, shorter provocation protocol, we cannot compare the results of the studies conducted by Atkins et al. (1990) and Pienkowski et al. (1988) with our results. Nevertheless, our results are analogical and lead to similar conclusions.

Though we claim the usefulness of our model of 9α,11β-PGF_2_ assessment with the use of skin chamber method for monitoring the reactivity of MC, we are aware that while interpreting the results of such a test in BVA patients, it is necessary to consider two factors: namely that (1) MC can be activated by venom components in a non-IgE-dependent manner and that (2) they can be degranulated due to mechanical stimuli. Taking into account that MC can be activated in a non-IgE dependent way by proteases and phospholipases of BV (e.g., via PAR2 receptor), to preclude the non-specific effect of phospholipase A_2_ on MC, we investigated parallels BV-sensitive and non-sensitive individuals. The fact that there is lack of a significant increase of 9α,11β-PGF_2_ concentration in non-allergic individuals lets us make an assumption that a significant 9α,11β-PGF_2_  increase in the BV allergic individuals was due to an allergen-specific effect of phospholipase A_2_. This seems to be the more likely since we used a very low concentration of BV. We decided to use venom concentration of 10^−4^ g/l, which is ten times smaller than the highest concentration recommended as the boundary diagnostic concentration in IDT. This decision was provoked by the concern of non-specific reactions that might be caused by 10^−3^ g/l concentration. Additionally, our experience with the skin chamber method endorses the lower concentration’s both diagnostic and safety value.

We also took into consideration the risk of MC degranulation due to vacuum effect, as a physical stimulus. Such mechanical activation of MC could be the reason for an increase of 9α,11β-PGF_2_ after 5 min of allergen and diluent provocation in both the groups of venom-sensitive and non-sensitive individuals. Considering this factor, to preclude the effect of the mechanical factor on the outcome of the study, the release of the mediators was calculated as a relation of the mediator concentrations in the allergen-challenged chamber to the mediator concentration in the allergen-free chamber.

While the fact of 9α,11β-PGF_2_ being detectable after 5 min in allergen-free chambers in the BV-allergic group can be accounted for by mechanical degranulation of MC caused by the suction action, the rise of 9α,11β-PGF_2_ after 15 min of diluent stimulation, and consequently lack of essential differences between 9α,11β-PGF_2_ levels at this time point in allergen-free and allergen-challenged chambers, remains unclear. We hypothesize that since the amount of mediators released from MC is proportionate to the density of MCs in the skin and is highly individualized, it is more appropriate to compare the dynamics of mediator release during allergen provocation rather than to compare the absolute quantified values of the released mediators. Additionally, it is possible that high 9α,11β-PGF_2_ levels in diluent chambers after 15 min result from the process of allergen absorption into blood circulation from the microvascular blister bed inside the allergen-stimulated chambers. This explanation is supported by the findings of Bochenek et al. (2004), who after bronchial challenge with specific allergen in a group of asthmatic patients sensitized to *D. pteronyssinus* observed significant increase of 9α,11β-PGF_2_ levels in plasma (*p* < 0.01) after 5 min following the provocation.

Analysis of ROC curve for 9α,11β-PGF_2_ ratio between 15th and 5th minute in allergen-challenged chamber showed better predictive properties of this parameter in discriminating between BVA patients and controls than random assessment. These properties are also better than reported by Cichocka-Jarosz et al. (2011), who performed ROC for plasma and urine PGD_2_ metabolites concentration in venom-allergic children. In our study, sensitivity of ratio of 9α,11β-PGF_2_ concentration between 15th and 5th minute in allergen-challenged chamber was equal to 71.4 %, while specificity reached 83.3 %, with the best area under the curve for 9α,11β-PGF_2_ equal to 74 % which are higher values than in Cichocka’s et al. (2011) study where the sensitivity of PG metabolites concentrations was lower than 70 %, while specificity not exceeded 55 %, with the best area under the curve for 9α,11β-PGF_2_ equal to 60 %.

Our study inscribes itself into a broader context of investigations of the processes of MC activation, contributing to the knowledge of the release of arachidonic acid metabolites under the allergen stimulation (Atkins et al. 1990; Bingham and Austin 2000; Bochenek et al. 2004; Shalit et al. 1988). We found out that 9α,11β-PGF_2_ is actively involved in the early allergic response to BV and can be measured during in vivo provocation with BV being potentially a marker useful for monitoring MC activation. Although the results of 9α,11β-PGF_2_ release measurements obtained in our study statistically differentiate BVA patients from healthy subjects, the method is not promising as a diagnostic instrument due to the results’ high inter-subject variability. Still, we believe, it might be used in the process of monitoring allergic patients, for instance, during VIT. Very interesting is the finding of extremely high response to allergen challenge in some patients, which has not been clinically reflected in the course of immunotherapy. This phenomenon requires further studies conducted in a larger group of patients with their long-term prospective observation.

## Abbreviations

IDT: Intradermal test
NADPH: Nicotinamide adenine dinucleotide phosphate
[2H] PGF_2α_: Deuterated prostaglandin F_2α_
MC: Mast cells
9α,11β-PGF_2_: Stereoisomer of prostaglandin F_2α_, prostaglandin F_2_ synthase product
ASA: Acetylsalicylic acid
BV: Bee venom
BVA: Bee venom allergy
PAR2 receptor: Protease activated receptor
PGD_2_: Prostaglandin D_2_
ROC: Receiver operator characteristics
Tetranor PGD-M: 9α-hydroxy-11,15-dioxo-13,14-dihydro-2,3,4,5-tetranor-prostan-1,20-dioic acid
VIT: Venom immunotherapy
